# A quantitative polymerase chain reaction-enzyme immunoassay for accurate measurements of human papillomavirus type 16 DNA levels in cervical scrapings

**DOI:** 10.1038/sj.bjc.6690659

**Published:** 1999-09

**Authors:** M V Jacobs, J M M Walboomers, J van Beek, F J Voorhorst, R H M Verheijen, C J L M Meijer, A J C van den Brule, ThJ M Helmerhorst, P J F Snijders

**Affiliations:** Departments of Pathology Section of Molecular Pathology, Epidemiology and Biostatistics, and Obstetrics and Gynecology, University Hospital Vrije Universiteit, 1081 HV, Amsterdam,The Netherlands

**Keywords:** HPV, Q-PCR-EIA, cervical scrapings

## Abstract

A quantitative polymerase chain reaction-enzyme immunoassay (Q-PCR-EIA) was developed to measure the amount of human papillomavirus (HPV) 16 DNA per genome equivalent in cervical scrapings. The quantitative approach was based on a combined competitive PCR for both HPV 16, using the general primer GP5+/6+ PCR, and β-globin DNA. The two competitive PCRs involve co-amplification of target sequences and exogenously added DNA constructs carrying a rearranged 30 bp sequence in the probe-binding region. The accuracy of quantification by combining the two competitive PCR assays was validated on mixtures of HPV 16 containing cervical cancer cells of CaSki and SiHa cell lines. Comparison of this fully quantitative PCR assay with two semi-quantitative HPV PCR assays on a series of crude cell suspensions from HPV 16 containing cervical scrapings revealed remarkable differences in the calculated relative HPV load between samples. We found evidence that correction for both intertube variations in PCR efficiency and number of input cells/integrity of DNA significantly influence the outcome of studies on viral DNA load in crude cell suspensions of cervical scrapings. Therefore, accurate measurements on viral DNA load in cervical scrapings require corrections for these phenomena, which can be achieved by application of this fully quantitative approach. © 1999 Cancer Research Campaign


*British Journal of Cancer* (1999) **81**(1), 114–121
© 1999 Cancer Research Campaign
Article no. bjoc.1999.0659

### A quantitative polymerase chain reaction-enzyme immunoassay for accurate measurements of human papillomavirus type 16 DNA levels in cervical scrapings

**MV Jacobs[1], JMM Walboomers[1], J van Beek[1], FJ Voorhorst[2], RHM Verheijen[3], CJLM Meijer[1], AJC van den Brule[1], ThJM Helmerhorst[3] and PJF Snijders[1]**

Departments of [1]Pathology, Section of Molecular Pathology, [2]Epidemiology and Biostatistics, and [3]Obstetrics and Gynecology, University Hospital Vrije Universiteit, De Boelelaan 1117, 1081 HV, Amsterdam, The Netherlands

**Summary** A quantitative polymerase chain reaction-enzyme immunoassay (Q-PCR-EIA) was developed to measure the amount of human papillomavirus (HPV) 16 DNA per genome equivalent in cervical scrapings. The quantitative approach was based on a combined competitive PCR for both HPV 16, using the general primer GP5+/6+ PCR, and β-globin DNA. The two competitive PCRs involve co-amplification of target sequences and exogenously added DNA constructs carrying a rearranged sequence in the probe-binding region. The accuracy of quantification by combining the two competitive PCR assays was validated on mixtures of HPV 16 containing cervical cancer cells of CaSki and SiHa cell lines. Comparison of this fully quantitative PCR assay with two semi-quantitative HPV PCR assays on a series of crude cell suspensions from HPV 16 containing cervical scrapings revealed remarkable differences in the calculated relative HPV load between samples. We found evidence that correction for both intertube variations in PCR efficiency and number of input cells/integrity of DNA significantly influence the outcome of studies on viral DNA load in crude cell suspensions of cervical scrapings. Therefore, accurate measurements on viral DNA load in cervical scrapings require corrections for these phenomena, which can be achieved by application of this fully quantitative approach.

**Keywords:** HPV; Q-PCR-EIA; cervical scrapings

To date, it is widely accepted that high-risk human papillomavirus types (HR-HPVs), particularly HPV 16 and 18, are causally involved in the development of cervical cancer and its precursor stages (IARC, 1995). An increasing prevalence of HR-HPV DNA has been found from low-grade to high-grade cervical intraepithelial neoplasia (CIN) lesions (Lörincz et al, 1992; Lungu et al, 1995), and HPV DNA is detectable in nearly all cervical carcinomas worldwide (Bosch et al, 1995; JMM Walboomers, personal communication).

Semi-quantitative PCR studies have shown that 45–96% of women with CIN III lesions contained a relatively high amount of DNA of at least one of the HR-HPV types 16, 18, 31, 33 and 35 in their cervical scrapings. In contrast, the majority (74–88%) of women with CIN I lesions displayed low HR-HPV DNA levels in their cervical scrapings (Cuzick et al, 1992, 1994; Bavin et al, 1993; Flanelly et al, 1995). These data are in favour of the viral load in cervical scrapings being an important determinant for severity of the underlying cervical lesion.

However, a study using the Hybrid Capture Assay (HCA), an HPV DNA detection technique not based on DNA amplification, has revealed lower amounts of viral DNA in cervical scrapings of women with CIN III lesions compared to these with CIN I lesions (Farthing et al, 1994). This was supported by Sun et al (1995), showing that only 30% of women with relatively high levels of HPV DNA in their cervical scrapings had underlying high-grade CIN.

*Received 29 September 1998*
*Revised 15 February 1999*
*Accepted 15 March 1999*

*Correspondence to:* PJF Snijders

The discrepancies between the different studies are likely to be due to the different methodologies used. Although all the methods used had in common a semi-quantitative approach, only in the PCR studies was a correction for the amount of input DNA made. Since it is conceivable that the number of cells varies considerably between cervical scrapings, it is likely that the HPV copy number values calculated from the HCA studies do not always reflect the actual viral load per genome equivalent. On the other hand, the semi-quantitative HPV PCR studies described thus far did not correct for eventual differences in amplification efficiency between different samples. This may be an additional factor affecting the outcome of studies on HPV DNA load.

Quantitation of housekeeping genes, most commonly β-globin or HLA genes, has been used successfully to correct for differences in input DNA between samples (Kellog et al, 1990). Moreover, to compensate for fluctuations in amplification efficiency between samples, exogenous DNA fragments have been incorporated in competitive PCR assays (Becker-André and Hahlbrock, 1989; Gilliland et al, 1990; Bouaboula et al, 1992; Mulder et al, 1994; Mallet et al, 1995). The exogenous DNA fragments (referred to as internal constructs) were often modified versions of the target DNA to ensure an equal amplification efficiency.

The technical limitations of previous studies on HPV DNA load prompted us in this study to develop a quantitative (Q-) PCR assay for accurate measurements of viral DNA load in cervical scrapings. Using an HPV general primer GP5+/6+-mediated PCR protocol (de Roda Husman et al, 1995) a competitive PCR assay based on the co-amplification of native HPV 16 target and its internal construct was developed. Similarly, a competitive β-globin gene PCR was developed. Following detection of PCR

**Table 1** Primer and probe sequence

| Primer | Target | Sequence (5′–3′) |
|---|---|---|
| (bio)$BGPCO_3$ | β-globin | ACACAACTGTGTTCACTAGC |
| $BGPCO_9$ | β-globin | CTTGTGGCCGCAAATCAGCAGGTCTTCCGATGTCTGTTTGAGGTT |
| $BGPCO_8$ | β-globin | TTGATACCAACCTGCCCAGG |
| $BGPCO_{10}$ | β-globin | TCGGAAGACCTGCTGATTTGCGGCCACAAGCTGCCGTTACTGCCC |
| GP16.5 | HPV 16 | TTTGTTACTGTTGTTGATACTAC |
| GP8 | HPV 16 | CAAGACGAAGAATAACGGATGCCTTTTGAATATTTGTACTGCTG |
| (bio)GP6+ | HPV 16 | GAAAAATAAACTGTAAATCATATTC |
| GP9 | HPV 16 | ATCAAAAGGCATCCGTTATTCTTCGTCTTGAACTACATATAAAAA |
| GP5+ | HPV 16 | TTTGTTACTGTGGTAGATACTAC |
| Probes | | |
| WTBGPRO | Endogenous β-globin | ACTTCTCCTCAGGAGTCAGGTGCACCATGG |
| ICBGPRO | β-globin internal construct | CTTGTGGCCGCAAATCAGCAGGTCTTCCGA |
| WTHPV16PRO | Endogenous HPV 16 | GTCATTATGTGCTGCCATATCTACTTCAGA |
| ICHPV16PRO | HPV 16 internal construct | ATCAAAGGCATCCGTTATTCTTCGTCTTG |

Underlined sequences represent the rearranged 30 base pairs probe binding sequence.

**Table 2** Accuracy of quantification by Q-PCR-EIA

| | Mean copy number of HPV 16 per cell | |
|---|---|---|
| No. of cells (total $10^5$ HPV 16 copies)* | Expected (min–max)[a] | Calculated (mean ± s.d.)[b] |
| 200 CaSki + 0 SiHa | 400–600 | 395 ± 168.5 |
| 195 CaSki + 500 SiHa | 113–175.5 | 94 ± 26.6 |
| 150 CaSki + 5000 SiHa | 12.6–27.2 | 11.5 ± 1.2 |
| 0 CaSki + 20 000 SiHa | 1–10 | 3.6 ± 0.7 |

[a]Based on the assumption that 1 SiHa cell contains 1–10 copies of HPV 16 and 1 CaSki cell contains 400–600 copies of HPV 16 (Caballero et al, 1995). [b]The determined values are averages of 3 independent experiments.

products by an enzyme immunoassay (EIA) (Jacobs et al, 1996, 1997), the ratio between outcomes of competitive HPV 16 and β-globin PCR was then used to determine the amount of HPV 16 DNA per genome equivalent. We demonstrated that co-amplification of internal construct and endogenous DNA of both targets was truly competitive and that this approach allowed for an accurate quantification of HPV 16 DNA on a per cell basis. Comparative analyses of this method and semi-quantitative PCR protocols on a series of HPV 16 containing cervical scrapings learned that correction for both amount/integrity of input DNA and differences in amplification efficiency may influence the outcome of studies on HPV DNA load. Consequently, in order to reconsider putative associations between viral load and clinical parameters this Q-PCR approach can be a potentially valuable tool.

### MATERIALS AND METHODS

### HPV 16 clone, human placental DNA, cell lines and clinical specimens

The pHPV 16 clone containing full-length HPV 16 DNA was kindly provided by H Zur Hausen and L Gissmann (Heidelberg, Germany). Human placental DNA was purchased from Sigma (USA).

The HPV 16 containing human cervical cancer cell lines SiHa and CaSki were obtained from the American Type Tissue Culture Collection.

For application of the Q-PCR assay, a total of 48 cytomorphologically abnormal cervical scrapings were used from women who had visited in the past the outpatients clinic of the University Hospital Vrije Universiteit in Amsterdam, The Netherlands. All 48 cervical scrapings contained only HPV 16 DNA as previously determined by HPV type-specific PCRs. The cervical scrapings were collected in 1 ml 10 mM Tris–HCl, pH 7.5 and prepared for PCR purposes as described earlier (van den Brule et al, 1990).

### Generation of internal constructs for HPV 16 and β-globin

Internal constructs were generated by site-directed mutagenesis with overlap extension using PCR as has been previously described (Rüster et al, 1995). The primer sequences used are shown in Table 1. Briefly, for β-globin primer combinations $BGPCO_3$/$BGPCO_9$ and $BGPCO_8$/$BGPCO_{10}$ and, for HPV 16, primer combinations GP16.5/GP8 and GP6+/GP9 were used to generate two PCR products with an overlapping, rearranged probe binding sequence at the 5′ end of the PCR products. The β-globin and HPV 16 PCR products were amplified from human placental DNA and pHPV 16 DNA respectively. The two overlapping PCR products were extended using $BGPCO_3$/$BGPCO_8$ and GP16.5/GP6+ primers for generating β-globin and HPV 16 constructs respectively. The PCR products were 140 base pairs for both targets. All PCRs were performed under conditions as

© 1999 Cancer Research Campaign

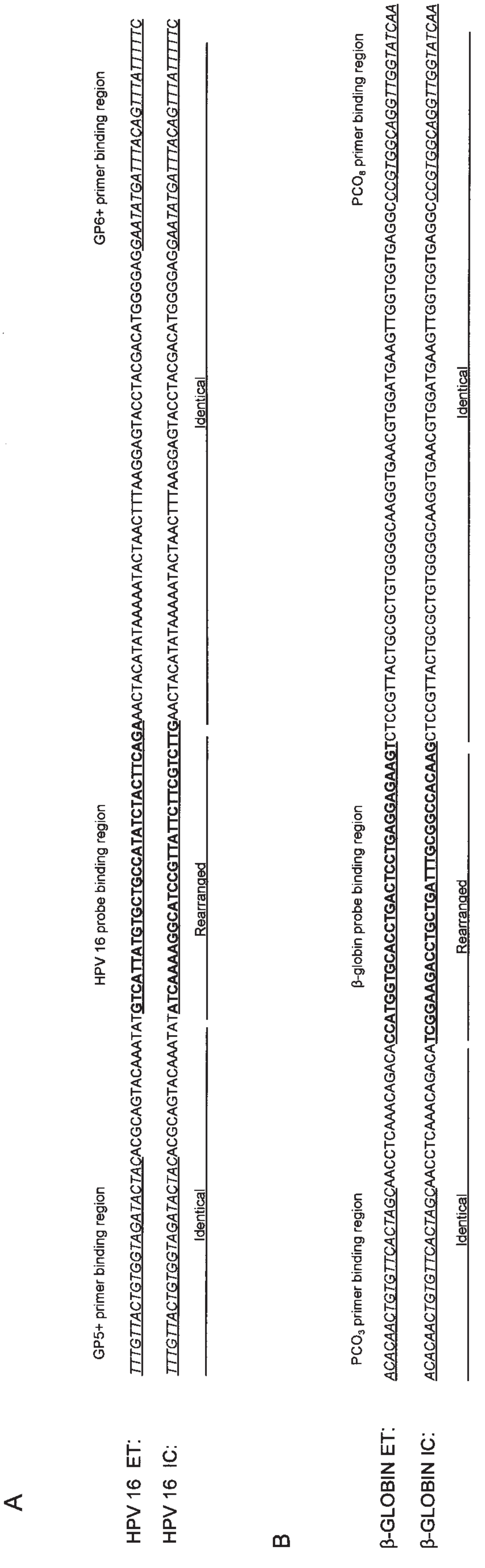

**Figure 1** Sequences of HPV 16 (**A**) and β-globin (**B**) endogenous templates (ET) and internal constructs (IC) that were generated by site-directed mutagenesis with overlap extension using PCR. *Italic* sequences represent primer binding region; **bold** sequences represent probe-binding region described for HPV type-specific PCR assays (van den Brule et al, 1990). Subsequently, the final PCR products were purified from a 1% agarose gel using a QIAEX II Gel Extraction kit (Qiagen, Germany) and cloned into a pCRScript Amp SK(+) vector (Stratagene, USA) following the instructions of the manufacturers' protocols. The cloned DNA fragments were then sequenced on a 373A automatic sequencer using dye terminators (Applied Biosystems, USA) to determine the cloned sequence. Subsequently, the concentration of the plasmids containing the cloned sequences was determined by spectrophotometric reading. Both clones were stored at –80°C until further use.

### Competitive PCR using internal constructs

For β-globin DNA, competitive PCRs were carried out in a total volume of 50 μl containing 50 mM potassium chloride (KCl), 10 mM Tris–HCl pH 8.3, 200 μM of each dNTP, 1.5 mM magnesium chloride ($MgCl_2$), 1 unit of Amplitaq DNA polymerase (Perkin-Elmer, Emmeryville, USA), 25 pmol of $BGPCO_3$ and $BGPCO_8$ primers, 10 μl of a sample and 10 μl of the β-globin internal construct of known concentration. The PCR cycling conditions were similar as referred to above.

Competitive PCRs for HPV 16 were performed with PCR mixtures consisting of 50 mM KCl, 10 mM Tris–HCl pH 8.3, 200 μM of each dNTP, 3.5 mM $MgCl_2$, 1 unit of Amplitaq DNA polymerase (Perkin-Elmer, Emmeryville, USA), 25 pmol of the general primers GP5+ (Table 1) and GP6+, 10 μl of the sample and 10 μl of the HPV 16 internal construct of known concentration. PCR amplification was performed under low stringency conditions as described elsewhere (de Roda Husman et al, 1995).

To enable detection of the co-amplified PCR-products in EIA, $BGPCO_3$ and GP6+ were biotinylated (bio$BGPCO_3$ and bioGP6+ respectively) at their 5′ end during synthesis (Eurogentec, Luik, Belgium). Samples were run in triplicate reactions, each spiked with a different concentration of the internal construct. From preliminary experiments it was learned that, in general, samples could be spiked with concentrations varying from $10^3$ to $10^5$ for HPV 16, and $10^4$ to $10^6$ for β-globin PCR. If the optical density (OD) ratio fell outside the 2-log range, new triplicate reactions were performed which were spiked with adjusted amounts of internal construct to ensure that OD ratios within a 2-log range were obtained. As positive controls, each internal construct dilution was amplified alone, while negative controls consisted of PCR mixtures without sample or construct added.

### EIA for the detection of PCR products

The PCR products were analysed in EIA as have been described previously (Jacobs et al, 1996, 1997). Briefly, biotinylated PCR products were captured on streptavidin-coated microtitre plates, denatured by alkaline treatment and hybridized to digoxigenin-11-ddUTP-labelled oligoprobes (Eurogentec, Luik, Belgium). Subsequent addition of anti-digoxigenin antibodies conjugated to alkaline phosphatase and p-nitrophenylphosphate as substrate finally enabled the detection of the ultimate hybrids by OD reading. The digoxigenin-labelled oligoprobes used are shown in Table 1.

### Quantitation of HPV 16 DNA load by Q-PCR-EIA

After competitive PCR, the amplification products were analysed in EIA, and the ODs generated by the internal constructs and the endogenous targets were measured for each individual reaction.

© 1999 Cancer Research Campaign

Assumed that one cell contains two copies of the β-globin gene the amount of HPV 16 DNA per genome equivalent was calculated according to the following formula: [(OD target$_{HPV\ 16}$/OD construct$_{HPV\ 16}$) × copies construct$_{HPV\ 16}$]:[(OD target$_{β\text{-globin}}$/OD construct$_{β\text{-globin}}$) × copies construct$_{β\text{-globin}}$)/2]. This formula was used provided that OD ratios fell within a 2-log range.

### Semi-quantitative, non-competitive PCR using external standards

For HPV 16 quantification by semi-quantitative, non-competitive PCR, aliquots of serial tenfold dilutions of pHPV 16 DNA ranging from 100 ag to 100 pg were amplified by GP5+/6+ PCR in parallel experiments with 10 µl of the crude cervical cell suspensions. PCR amplifications were carried out as described by de Roda Husman et al (1995).

Following amplification, a standard curve was generated by plotting the optical densities of the PCR products amplified from the dilutions of pHPV 16 DNA against the input amount of these amplified standards. To determine the initial amount of HPV 16 DNA in a given sample, the HPV copy numbers were deduced from the generated standard curve (Jacobs et al, 1996).

In addition, sample preparation, preparation of PCR solutions, spiking of the internal constructs and amplification/analysis of the PCR products were performed in four physically separated rooms under strict laboratory discipline to avoid contamination. Moreover, Q-PCR reactions were carried out with the same primer-, internal construct- and probe batches, whereas analysis of the different PCR products of each sample was performed in the same EIA microtitre plate to minimize inter-assay variation.

### Statistical analysis

Direct comparison of the results obtained by the different (semi)-quantitative HPV PCR methods is not possible due to the use of different measurement units in which viral load is expressed (fg ml$^{-1}$ vs fg cells$^{-1}$). Therefore, statistical analysis was based on ranking of values. The Spearman rank correlation coefficient ($r_s$) was calculated to assess the change in association between samples measured by different (semi-)quantitative HPV PCR methods. A $P$-value < 0.05 indicates that the difference between the two correlation coefficients is significant (Snedecor and Cochran, 1980).

### RESULTS

### Assay strategy and generation of internal constructs

In order to develop a quantitative test that accurately assess the viral load in cervical scrapings, we used the strategy of two competitive PCRs for quantification of HPV 16 DNA and β-globin DNA respectively. The ratio of the outcomes of the competitive PCRs for HPV 16 and β-globin DNA enables a calculation of the HPV 16 DNA load per genome equivalent. For the competitive HPV PCR, the general primer GP5+/6+-mediated PCR system (de Roda Husman et al, 1995) was chosen since it allows for the detection of all major HPV types (HPV 16, 18, 45, 51, 52, 58 and 59) which are considered currently carcinogenic for humans on the basis of case–control studies (Chichareon et al, 1997; Ngelangel et al, 1997). However, since HPV 16 is most commonly found in cervical carcinomas (Bosch et al, 1995), we first focused on the development of a competitive PCR for this HPV type.

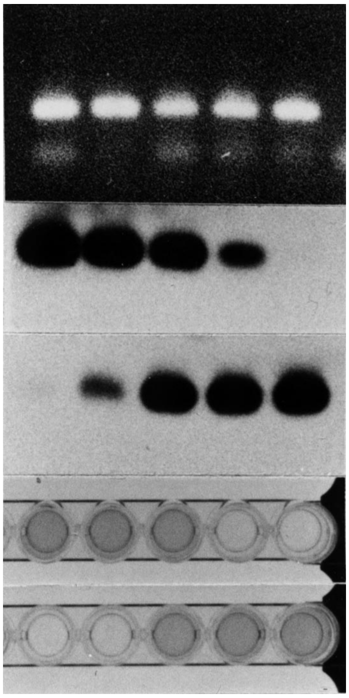

**Figure 2** Competitive PCR results of endogenous HPV 16 target and HPV 16 internal construct. Lane 1: 10 pg pHPV 16 DNA + 10$^2$ copies of internal construct; lane 2: 1 pg pHPV 16 DNA + 10$^3$ copies of internal construct; lane 3: 100 fg pHPV 16 DNA + 10$^4$ copies of internal construct; lane 4: 10 fg pHPV 16 DNA + 10$^4$ copies of internal construct; lane 5: 1 fg pHPV 16 DNA + 10$^5$ copies of internal construct. PCR products are shown after gel analysis (**A**), and additional Southern blot hybridization of the PCR products with either a specific oligoprobe for HPV 16 endogenous template (**B**) or HPV 16 internal construct (**C**). PCR products were also differentiated in EIA using oligoprobes for the target template (**D**) and internal construct (**E**) respectively

The internal constructs for this approach were made by site-directed mutagenesis. These constructs represent HPV 16 and β-globin gene sequences, including the binding sites for GP5+/6+ and $PCO_3$/$PCO_8$ primer combinations respectively. Although they give rise to PCR products of the same size as the native targets, the constructs differ in sequence from the targets by a rearranged intervening 30 bp sequence (Figure 1). Following PCR, these rearranged sequences enables distinguising PCR products derived from the internal constructs from those of the target sequences by specific oligo probes targeting this region.

### Competition of amplification between endogenous templates and internal constructs in PCR

A reliable competitive PCR assay requires the internal constructs to be co-amplified with the same efficiency as the target DNA. Therefore, it was first tested whether competition of amplification between targets and corresponding internal constructs is indeed equal. For this purpose, serial tenfold dilutions of target DNA, in which the amount of endogenous template ranged from 1 to 10$^8$ copies, were spiked with serial tenfold dilutions (10$^8$ to 1 copies) of the internal constructs into opposite directions. After competitive PCRs, the amplification products were analysed by gel electrophoresis, followed by Southern blot hybridization (Jacobs et al, 1996), and by EIA respectively. As shown in Figure 2A for HPV

*© 1999 Cancer Research Campaign* 

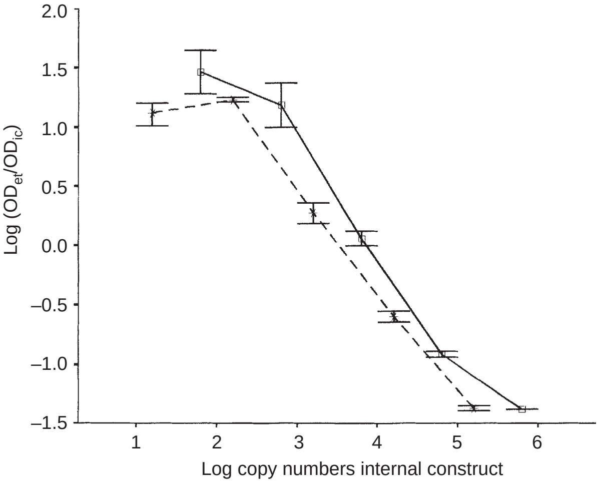

**Figure 3** Analysis of HPV 16 and β-globin PCR-EIA. The ratio of optical densities (ODs) from endogenous template to internal construct ($OD_{ET}/OD_{IC}$) is plotted on a log-scale against the concentration of the internal construct. The median values and standard deviations are shown. (——) HPV 16, slope $P = -1.08$ in the range of log values between –1 and +1; (- - - -) β-globin, slope $P = -0.89$ in the range of log values between –1 and +1

16, agarose gel analysis revealed PCR products of the same size derived from both target and internal construct. Moreover, equal input amounts of target and internal construct resulted in PCR products with the same signal intensities after Southern blot hybridization (Figure 2B,C) and equal OD values in EIA (Figure 2D,E) after hybridization with target versus construct probes. This suggests a true competitive feature of the assay. Thus, when the amounts of PCR products derived from target and internal construct are the same, this indicates that quantities of target DNA are identical to those of spiked construct.

To investigate to what extent quantification is possible when the amount of initial internal construct differs from target, the HPV 16 target/construct OD ratio values calculated from the competitive PCR-EIA experiment described in Figure 2D and 2E were expressed as function of the input amounts of internal construct. As shown in Figure 3, the curve obtained showed a linear decrease of target/construct OD ratio with increasing concentration of internal construct within the range of 0.1–10. Since within this 2-log range the slope of the curve was very close to −1, this indicates indeed that both target and construct were amplified with equal efficiencies. Similar results were obtained from competitive β-globin gene PCR assays (Figure 3). The fact that linearity was only evident when the OD ratios ranged from 0.1 to 10 indicates that a reliable quantification requires that the amount of spiked construct does not differ more than 2-log from the amount of target DNA. In practice, this requires samples to be spiked with several amounts of internal construct in parallel experiments to meet this criterion.

### Detection limit and accuracy of Q-PCR-EIA

In order to find out to what level the competitive approach influences the sensitivity of detecting target DNA, serial tenfold dilutions of SiHa DNA containing approximately 1–10 HPV 16 copies per cell and human placental DNA were spiked with different amounts of HPV 16 and β-globin internal constructs, respectively, and subjected to Q-PCR-EIA. It appeared that the detection limit of HPV 16

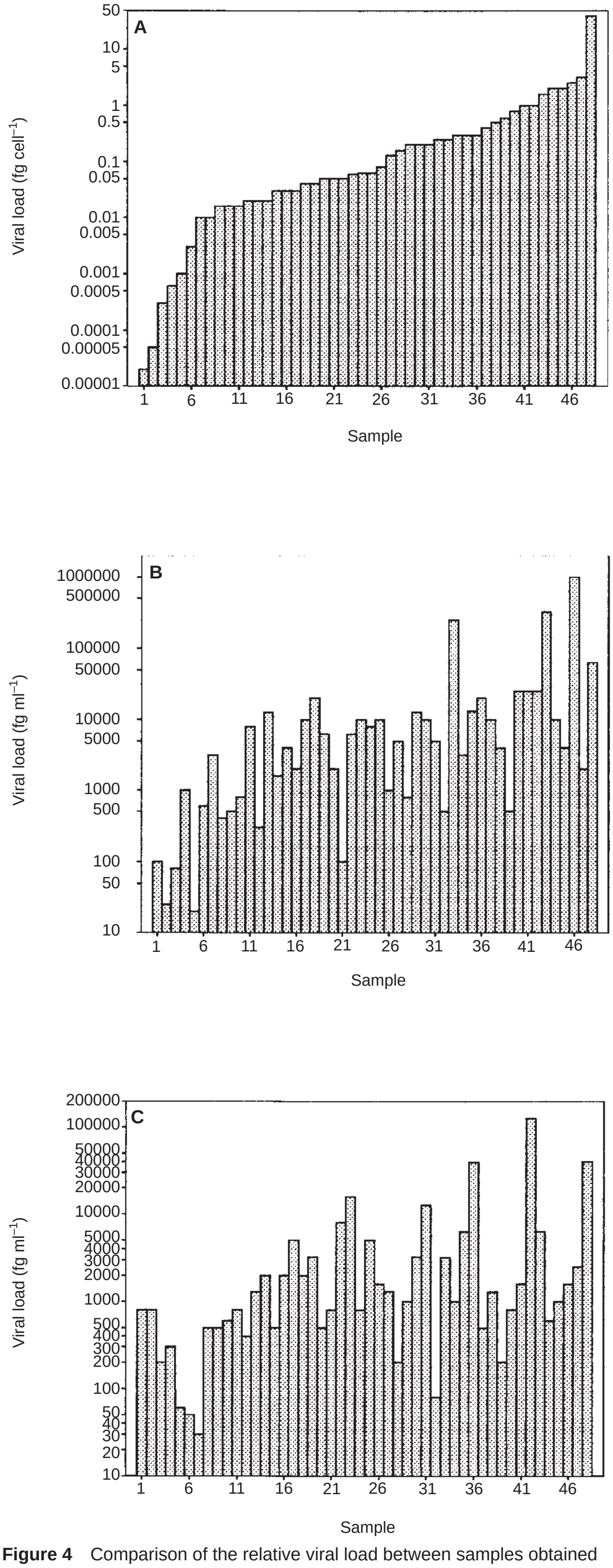

**Figure 4** Comparison of the relative viral load between samples obtained with quantitative PCR-EIA and two semi-quantitative approaches. Each column on the *x*-axis represents an individual sample. Samples are ordered according to increasing viral load values as calculated with the fully quantitative Q-PCR-EIA. (**A**) Competitive GP5+/6+ PCR combined with competitive β-globin PCR (Q-PCR-EIA); (**B**) competitive GP5+/6+ PCR without correction for β-globin quantities; (**C**) semi-quantitative non-competitive GP5+/6+ PCR

© 1999 Cancer Research Campaign

Q-PCR-EIA was around 1 ng SiHa DNA which corresponds to $10^3$–$10^4$ copies of HPV 16 DNA. For β-globin Q-PCR-EIA the detection limit was around 1 ng of human placental DNA corresponding to around 400 copies of the β-globin gene. This indicates that by combining the two competitive PCR assays, quantification is possible provided that at least $10^3$–$10^4$ copies of HPV 16 and 400 copies of β-globin gene are present in the sample.

Subsequently, the accuracy of quantification by Q-PCR-EIA was tested on four mixtures of cells from the cervical cancer cell lines SiHa and CaSki (containing approximately 500 HPV 16 copies per cell). For this purpose, different amounts of SiHa and CaSki cells were mixed in such a way that the samples contained a total of about $10^5$ copies of the HPV 16 DNA. However, the mean HPV 16 copy number per cell varied considerably between the different mixtures from approximately 500 to 5 copies per cell. As shown in Table 2, the number of HPV 16 copies per genome equivalent as measured with Q-PCR-EIA approximated the expected mean HPV 16 copy number per cell for all four dilutions analysed. It should be noted that the standard deviation decreased with increasing number of cells used for the PCR, suggesting that the reproducibility of the assay increased with increasing number of cells present in the assay.

### Comparison between quantitative PCR and semi-quantitative PCR assays on cervical scrapings

Finally, Q-PCR-EIA was applied to 48 HPV 16 containing cervical scrapings and the outcome was compared with two less stringent PCR approaches, i.e. a semi-quantitative non-competitive PCR-EIA (Jacobs et al, 1996, 1997) and the competitive GP5+/6+ PCR-EIA for HPV 16 without correction for housekeeping gene (i.e. β-globin gene) quantities. Relative quantities of HPV 16 DNA load calculated by the three methods are shown in Figure 4.

Comparison of the amounts of HPV 16 copy numbers calculated by the competitive HPV GP5+/6+ PCR combined with competitive β-globin PCR and the competitive HPV GP5+/6+ PCR only (Figure 4A vs 4B) using the Spearman rank correlation test revealed a correlation coefficient ($r_s$) of 0.63 (95% confidence interval (CI) 0.42–0.78). Comparison of the HPV 16 DNA loads measured by the competitive HPV GP5+/6+ PCR and the semi-quantitative non-competitive GP5+/6+ PCR (Figure 4B vs 4C) yielded a $r_s$ of 0.67 (95% Cl 0.47–0.80). The difference between the two $r_s$ values was not significant ($P = 0.638$). These results indicate clear differences in the assessment of the viral load in cervical scrapings depending on the particular method used.

### DISCUSSION

Several studies have shown that the number of HPV DNA copies in cervical scrapings may be predictive for the severity of underlying CIN lesion (Cuzick et al, 1992, 1994; Bavin et al, 1993; Flanelly et al, 1995). In addition, it has also been shown that high HPV DNA load in cervical scrapings is associated with persistent CIN (Ho et al, 1995). Hence, HPV DNA load in cervical scrapings may be a valuable marker, not only for the severity of underlying cervical lesions but also for the prediction of persistent CIN. Therefore, methods allowing accurate quantification of viral DNA in cervical scrapings could be of value to distinguish high-grade and/or persistent CIN.

In this study we developed a quantitative HPV PCR assay (Q-PCR-EIA) which corrects both for intertube variations in PCR efficiency and for variability in the amount of input DNA. This assay would therefore be useful to quantitate HPV DNA in crude suspensions of cervical scrapings. Ultimately, viral levels could be expressed as copy numbers per genome equivalent by calculating HPV 16/β-globin PCR-EIA OD ratios obtained after co-amplification of target DNA and an exogenously added internal construct. The results demonstrated that this Q-PCR-EIA approach provides a reliable indication of the viral load in cervical scrapings.

Important for a reliable quantification by competitive PCR assays is a similar amplification efficiency between target DNA and competitor (Ferre, 1992). To increase the likelihood that both endogenous and exogenous templates will be amplified with equal efficiencies, internal constructs were designed in such a way that primer binding sequences, sizes and base composition were similar compared to the target sequences. Application of these constructs in competitive PCR assays revealed an equal amplification efficiency as long as the target/construct OD ratio fell within a 2-log range. Such a dynamic range is comparable to that of other competitive PCR assays developed for a variety of micro-organisms (Kellog et al, 1990; Bouaboula et al, 1992; Mallet et al, 1995; Rüster, Zeuzem and Hahlbrock, 1995; Rowe et al, 1997).

The HPV 16 Q-PCR-EIA was validated in reconstruction experiments using mixtures of two different cervical carcinoma cell lines (CaSki and SiHa) with known HPV 16 copy number per cell. The experiments revealed a good agreement between expected and calculated mean viral copy numbers per genome. The lack of exact agreement may be due to slight variations in the exact HPV 16 copy numbers in CaSki and/or SiHa cells, or, alternatively aneuploidy of the cancer cells. The latter may result in an altered mean copy number of the β-globin gene per cell. Moreover, it has to be noted that the reproducibility (standard deviation) of the quantitative method was markedly lower for the dilutions containing only 200 cancer cells. This is most likely due to the limit of quantification by the competitive β-globin PCR which was found to be at the level of 400 copies of the β-globin gene, equivalent to 200 diploid cells. However, it is more conceivable that in practice the threshold of the competitive HPV 16 PCR ($10^3$–$10^4$) is the limiting factor for the quantitative approach since cervical scrapings contain on average $10^5$–$10^6$ exfoliated cells (Lörincz, 1996).

The comparison of different (semi-)quantitative HPV PCR assays on a series of HPV 16 positive cervical scrapings revealed that two major factors affect the outcome of studies on quantitative HPV DNA load. First, the observed difference between competitive HPV GP5+/6+ PCR and a combined competitive HPV/β-globin PCR indicates the existence of variations in the number of cells present in cervical scrapings and/or integrity of DNA. Second, an additional difference in relative viral load was evident after comparison of competitive GP5+/6+ PCR and the semi-quantitative non-competitive GP5+/6+ PCR. The latter points to intertube variations in PCR efficiency. Apparently, cervical scrapings differ considerably with respect to the eventual presence and amount of PCR interfering factors. Since the calculated $r_s$ values did not differ significantly, it can be concluded that both intertube variations in PCR efficiency and variations in amounts/integrity of input DNA equally affect quantitative measurements of HPV DNA in cervical scrapings.

Previously, Kinoshita et al (1993) have described a competitive HPV 16 PCR based on primer sequences from the E6 region. However, their approach did not include a correction for the amount of input DNA. Therefore, this method is only applicable to constant input amounts of isolated DNA, rather than crude cell

*© 1999 Cancer Research Campaign*

suspensions. Still, even this approach is hampered by the fact that no corrections are made for eventual variations in integrity of the DNA between different DNA samples. Another, more simple approach has been described by Caballero et al (1995). Their method has been based on differences in signal intensities between HPV-specific and co-amplified non-specific DNA of higher molecular weight, generated in a low-stringency PCR system using HPV general primers GP5/6. In this assay, the amplified non-specific products serve as controls for both PCR efficiency and amounts/integrity of input DNA. A major advantage of this method is that only a single PCR reaction is necessary to meet all criteria to be fulfilled for reliable quantification. However promising, this method is subject to limitations as well. In our hands, efficiency of HPV 16 DNA amplification was not proportional to the amplification efficiency of the non-specific fragment in dilution experiments (data not shown), particularly when the levels of HPV target were relatively high. This suggests a non-equal competition between amplification of both targets, which may partly be due to the fact that the non-specific fragment is larger in size. Moreover, the non-specific target of unknown origin may not match as good with the PCR primers as the HPV target. This also has the consequence that minor variations in stringency, due to differences between crude cell suspensions, may have a more drastic affect on the efficiency of amplifying non-specific target than HPV target. Therefore, we feel that this method is more sensitive to subtle variations in PCR conditions, which may particularly be the case when using crude cervical samples rather than isolated DNA. In contrast, the Q-PCR-EIA described in this study is completely independent from slight differences in PCR conditions and would be the method of choice for crude cell suspensions. Since this method is based on the general primer format, it potentially can be converted to Q-PCR assays for other HPV types. Of course, such a conversion requires first additional reconstruction experiments to test the efficiency of competition before application in practice.

Finally, this Q-PCR-EIA system provides a valuable tool to reconsider putative associations between viral load and clinical parameters. In this context, we are currently investigating HPV 16 DNA load in a non-intervention follow-up study of women with abnormal cytology (Remmink et al, 1995). The outcome of such studies ultimately will clarify whether or not viral load has any predictive value for persistent viral infections and/or severity of underlying CIN lesions.

### ACKNOWLEDGEMENTS

We are grateful to Nathalie Fransen-Daalmeijer, René Pol, Rick van Andel and Henri Schrijnemakers for technical assistance. This work was partly supported by a grant from the Prevention Fund, The Netherlands (Grant no. 28-1502,2).

### REFERENCES

Bavin PJ, Giles JA, Deery A, Crow J, Griffiths PD, Emery VC and Walker PG (1993) Use of semi-quantitative PCR for human papillomavirus DNA type 16 to identify women with high-grade cervical disease in a population presenting with a mild dyskaryotic smear report. *Br J Cancer* **67**: 602–605

Becker-André M and Hahlbrock K (1989) Absolute mRNA quantification using the polymerase chain reaction (PCR). A novel approach by a PCR aided transcript titration assay (PATTY). *Nucleic Acids Res* **17**: 9437–9446

Bosch FX, Manos MM, Muñoz N, Sherman M, Jansen AM, Peto J, Schiffman MH, Moreno V, Kurman R, Shah KV and the IBSSC Study Group (1995) Prevalence of human papillomavirus in cervical cancer: a worldwide perspective. *J Natl Cancer Inst* **87**: 796–802

Bouaboula M, Legoux P, Pességué B, Delpech B, Dumont X, Piechaczyk M, Casellas P and Shires D (1992) Standardization of mRNA titration using a polymerase chain reaction method involving co-amplification with a multispecific internal control. *J Biol Chem* **267**: 21830–21838

Caballero OL, Villa LL and Simpson AJG (1995) Low stringency-PCR (LS-PCR) allows entirely internally standardized DNA quantitation. *Nucleic Acids Res* **23**: 192–193

Chichareon S, Herrero R, Muñoz N, Bosch FX, Jacobs MV, Deacon J, Santamaria M, Chongsuvivatwong V, Meijer CJLM and Walboomers JMM (1997) Risk factors for cervical cancer in Thailand: a case-control study. *J Natl Cancer Inst* **90**: 43–49

Cuzick J, Terry G, Ho L, Hollingwoth T and Anderson M (1992) Human papillomavirus type 16 DNA in cervical smears as predictor of high-grade cervical cancer. *Lancet* **339**: 959–960

Cuzick J, Terry G, Ho L, Hollingwoth T and Anderson M (1994) Type-specific human papillomavirus DNA in abnormal scrapings as a predictor of high-grade cervical intraepithelial neoplasia. *Br J Cancer* **69**: 167–171

de Roda Husman AM, Walboomers JMM, van den Brule AJC, Meijer CJLM and Snijders PJF (1995) The use of general primers GP5 and GP6 elongated at their 3′ ends with adjacent highly conserved sequences improves human papillomavirus detection by PCR. *J Gen Virol* **76**: 1057–1062

Farthing A, Masterson P and Vousden KH (1994) Human papillomavirus detection by hybrid capture and its possible clinical use. *J Clin Pathol* **47**: 649–652

Ferre F (1992) Quantitative or semi-quantitative PCR: reality versus myth. *PCR Meth Appl* **2**: 1–9

Flanelly G, Jiang G, Anderson D, Melvin W, Mann E and Kitchener H (1995) Serial quantitation of HPV 16 in the smears of women with mild and moderate dyskaryosis. *J Med Virol* **4**: 6–9

Gilliland G, Perrin S, Blanchard K and Bunn HF (1990) Analysis of cytokine mRNA and DNA: detection and quantification by competitive polymerase chain reaction. *Proc Natl Acad Sci USA* **87**: 2725–2729

Ho GYF, Burk RD, Klein S, Kadish AS, Chang CJ, Palan P, Bassu J, Tachezy R, Lewis R and Romney S (1995) Persistent genital human papillomavirus infection as a risk factor for persistent cervical dysplasia. *J Natl Cancer Inst* **18**: 1365–1371

IARC (1995) *Monographs on the Evaluation of Carcinogenic Risks to Humans. Human Papillomaviruses, Vol. 64, Human Papillomaviruses.* International Agency for Research on Cancer: Lyon

Jacobs MV, van den Brule AJC, Snijders PJF, Helmerhorst Th, Meijer CJLM and Walboomers JMM (1996) A non-radioactive PCR-enzyme immunoassay enables a rapid identification of HPV 16 and 18 in cervical scrapes after GP5+/6+ PCR. *J Med Virol* **49**: 223–229

Jacobs MV, Snijders PJF, van den Brule AJC, Helmerhorst ThM, Meijer CJLM and Walboomers JMM (1997) A general primer GP5+/GP6+-mediated PCR-enzyme immunoassay method for rapid detection of 14 high-risk and 6 low-risk human papillomavirus genotypes in cervical scrapings. *J Clin Microbiol* **35**: 791–795

Kellog DE, Sninsky JJ and Kwok S (1990) Quantitation of HIV-1 proviral DNA relative to cellular DNA by the polymerase chain reaction. *Anal Biochem* **189**: 202–208

Kinoshita M, Shin S and Aono T (1993) A sensitive and quantitative method for determination of number of HPV 16 DNA copies by using the competitive polymerase chain reaction. *GATA* **10**: 161–121

Lörincz A (1996) Hybrid Capture method for detection of human papillomavirus DNA in clinical specimens. *Papillomavirus Report* **7**: 1–5

Lörincz AT, Reid R, Jenson AB, Greenberg MD, Lancaster W and Kurman RJ (1992) Human papillomavirus infection of the cervix: relative risk associations of 15 common anogenital types. *Obstet Gynecol* **79**: 328–337

Lungu OX, Sun XW, Wright TC, Ferenczy A, Richart RM and Silverstein S (1995) A polymerase chain reaction-enzyme-linked immunosorbent assay method for detecting human papillomavirus in cervical carcinomas and high-grade cervical cancer precursors. *Obstet Gynecol* **85**: 337–342

Mallet F, Herbrard C, Livrozet JM, Lees O, Tron F, Touraine JL and Mandrand B (1995) Quantitation of human immunodeficiency virus type 1 DNA by two PCR procedures coupled with enzyme-linked oligosorbent assay. *J Clin Microbiol* **12**: 3201–3208

Mulder J, McKinney N, Christopherson C, Sninsky J, Greenfield L and Kwok S (1994) Rapid and simple PCR assay for quantitation of human immunodeficiency virus type 1 RNA in plasma: application to acute retroviral infection. *J Clin Microbiol* **2**: 292–300

Ngelangel C, Muñoz N, Bosch FX, Limson GM, Festin MR, Deacon J, Jacobs MV, Santamaria M, Meijer CJLM and Walboomers JMM (1997) The causes of cervical cancer in the Philippines: a case-control study. *J Natl Cancer Inst* **90**: 50–57

© 1999 Cancer Research Campaign

Remmink AJ, Walboomers JMM, Helmerhorst ThJM, Rozendaal L, Risse EKJ, Meijer CJLM and Kenemans P (1995) The presence of persistent high-risk human papillomavirus genotypes in dysplastic cervical lesions is associated with pregressive disease: natural history up to 36 months. *Int J Cancer* **61**: 306–311

Rowe DT, Qu L, Reyes J, Jabbour N, Yunis E, Putman P, Todo S and Green M (1997) Use of quantitative competitive PCR to measure Epstein-Barr virus genome load in the peripheral blood of pediatric transplant patients with lymphoproliferative disorders. *J Clin Microbiol* **6**: 1612–1615

Rüster B, Zeuzem S and Roth WK (1995) Quantification of hepatitis C virus RNA by competitive reverse transcription and polymerase chain reaction using a modified hepatitis C virus RNA transcript. *Anal Biochem* **224**: 597–600

Snedecor GW and Cochran WG (1980) *Statistical methods.* Chapter 10, Correlation, pp 175–193. The lowa State University Press, Amnes, Iowa, U.S.A.

Sun X-W, Ferenczy A, Johnson D, Koulos JP, Lungu O, Richart RM and Wright TC (1995) Evaluation of the Hybrid Capture human papillomavirus deoxyribonucleic acid detection test. *Am J Obstet Gynecol* **3**: 1432–1437

van den Brule AJC, Meijer CJLM, Bakels V, Kenemans P and Walboomers JMM (1990) Rapid human papillomavirus detection in cervical scrapes by combined general primer-mediated and type-specific polymerase chain reaction. *J Clin Microbiol* **28**: 2739–2743

Wang M, Doyle MV and Mark DF (1989) Quantitation of mRNA by the polymerase chain reaction. *Proc Natl Acad Sci* **86**: 9717–9721

*© 1999 Cancer Research Campaign*
